# Factors influencing real time internal structural visualization and dynamic process monitoring in plants using synchrotron-based phase contrast X-ray imaging

**DOI:** 10.1038/srep12119

**Published:** 2015-07-17

**Authors:** Chithra Karunakaran, Rachid Lahlali, Ning Zhu, Adam M. Webb, Marina Schmidt, Kyle Fransishyn, George Belev, Tomasz Wysokinski, Jeremy Olson, David M. L. Cooper, Emil Hallin

**Affiliations:** 1 Canadian Light Source Inc., 44 Innovation Boulevard, Saskatoon, SK, Canada S7N 2V3; 2 University of Saskatchewan, 110 Science Place, Saskatoon, SK, Canada S7N 5C9; 3 Department of Anatomy and Cell Biology, University of Saskatchewan, 107 Wiggins Road, Saskatoon, SK, Canada S7N 5E5

## Abstract

Minimally invasive investigation of plant parts (root, stem, leaves, and flower) has good potential to elucidate the dynamics of plant growth, morphology, physiology, and root-rhizosphere interactions. Laboratory based absorption X-ray imaging and computed tomography (CT) systems are extensively used for *in situ* feasibility studies of plants grown in natural and artificial soil. These techniques have challenges such as low contrast between soil pore space and roots, long X-ray imaging time, and low spatial resolution. In this study, the use of synchrotron (SR) based phase contrast X-ray imaging (PCI) has been demonstrated as a minimally invasive technique for imaging plants. Above ground plant parts and roots of 10 day old canola and wheat seedlings grown in sandy clay loam soil were successfully scanned and reconstructed. Results confirmed that SR-PCI can deliver good quality images to study dynamic and real time processes such as cavitation and water-refilling in plants. The advantages of SR-PCI, effect of X-ray energy, and effective pixel size to study plant samples have been demonstrated. The use of contrast agents to monitor physiological processes in plants was also investigated and discussed.

Internal structural visualization and quantification of plant parts are essential to fully understand the structure-function relationship and dynamic processes in plants. Knowledge of internal structures of plants is crucial for better understanding of morphological diversity, physiology, function, and evolution. Internal structural characterization of plants on the micron to nanometre scales is usually achieved by analyzing excised cross sections using optical light microscopy (LM), transmission electron microscopy (TEM), scanning electron microscopy (SEM) or confocal laser scanning microscopy (CLSM). These techniques are limited by sampling area, accurate sample location, number of analyzed samples, and extensive sample preparation protocols, all of which can make data interpretation more difficult. The dynamic processes like water or nutrient flow and long term continuous monitoring of plants to understand physiological changes or responses to abiotic, biotic, or nutritional stresses are more easily studied with less invasive techniques.

The light and electron microscopy techniques produce good quality images at high spatial resolution. However, they are largely restricted to two dimensional (2D) imaging. SEM allows visualization and study of surface characteristics of plants or plant structures^1^ not only at high spatial resolution (~2 nm), but also as three dimensional (3D) objects. With an environmental SEM (ESEM), many biological samples can be investigated in their natural hydrated state^2^. CLSM has been effectively used to visualize cellular structures as well as to produce 3D images of larger anatomical structures of plant tissues with high resolution^3^,^4^,^5^, including visualization of endophytic microorganisms in plant roots^6^ using fluorescent dyes^7^. These microscopic techniques require a variety of time consuming sample preparation methods and staining agents to improve differentiation of specific sample features^8^. The extensive sample preparation methods may alter morphologies and produce artefacts.

The use of X-rays to study opaque and thick agricultural samples started in the 1920s and 3D visualization of structures using X-rays—computed tomography (CT) was realized and demonstrated in 1973^9^. X-rays from synchrotrons (SR) have unique properties such as high flux density (photons/s/mm^2^), selective wavelength, and partial coherence compared to X-rays from laboratory X-ray machines. High flux density simultaneously reduces exposure times and improves the signal to noise ratio (SNR). Wavelength tunability allows the use of the optimal wavelength for the plant component being imaged (*in situ* roots or above ground soft tissues are better imaged with different wavelengths). The partial coherence of the SR X-ray beam has unique advantages to image low density tissues that have weak absorption or interactions with X-rays^10^,^11^.

Some of the common SR based X-ray imaging modalities are absorption imaging, phase contrast imaging (PCI), and diffraction enhanced imaging (DEI). X-rays typically interact with samples by being absorbed or scattered. Absorption X-ray imaging is based on sample density differences whereas PCI is based on the refraction of X-rays. The X-ray absorption and phase contrast (PC) images reveal morphological features of dry plant samples at sub-micrometer spatial scale^12^,^13^. The PCI highlights small changes within a sample by producing edge enhanced images^14^. For example, at high X-ray energies (~>5 keV), the phase signal by low density materials is about 3 orders higher than the absorption signal^15^,^16^. Further, PCI is more sensitive to phase gradients than DEI^15^. In the study showing the application of DEI for plant sciences, samples had to be kept inside water to minimize surface diffraction effects and hence it is not easy to image live plants^17^. DEI is good for thick samples (e.g. acrylic plastic of 36 mm thick and 40 to 45 mm thick compressed breast tissue) and DEI rejects scattering from samples and hence produces images with better contrast than absorption imaging^18^. X-ray absorption and in-line PCI share simple setup and similar data collection times, however, the sample to detector distance is larger in the case of PCI. DEI requires complex set up and the data acquisition time is about three times higher than PCI^18^. For these reasons, SR-PCI was selected as the best candidate technique to study low density light element plant tissues, especially for events like cavitation.

The main requirements for PCI are highly parallel or coherent X-ray beam and large sample to detector distance compared to absorption imaging^19^. Some laboratory PCI systems are being developed using micron size sources; however, they suffer from relatively low flux. Recently, SR-PCI has been shown to be an attractive, fast, and real-time minimally invasive technique for visualization of complex internal structures of plants and animals^20^,^21^. This technique has been successfully and extensively used in medical imaging^22^,^23^,^24^ and is still underutilized in agricultural sciences^20^. Developing fast and minimally destructive techniques to visualize, understand, and quantify internal structures of complex plant parts such as roots, stem, flowers, and rhizosphere is still challenging, and requires further work and development. Most of the reported works in the literature on the use of SR-PCI in plants have used cut and dried plant parts^25^,^26^ and artificial soil medium^27^. Therefore, the objectives of this work using an SR X-ray source were to: 1) compare absorption imaging and PCI of live plant parts; 2) determine the optimum X-ray energy for SR-PCI of seedlings *in vivo*; 3) determine the optimum effective pixel size required for plant imaging using PCI; 4) determine the best soil type to image plant roots *in vivo*; and 5) explore the use of contrast agents to monitor physiological and dynamic processes in live plants.

## Results

### SR-PCI vs absorption imaging

**Figure 1:**
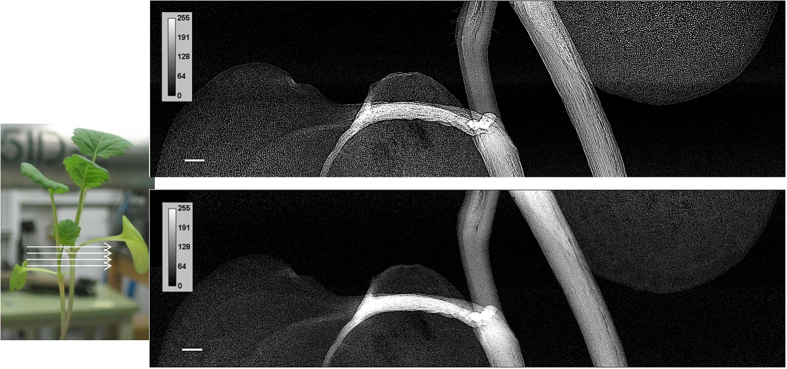
Advantages of phase contrast X-ray imaging (top, sample to detector distance ~85 cm) of plants compared to absorption X-ray imaging (bottom, sample to detector distance ~8.5 cm). X-ray energy used = 18 keV, exposure time = 1 s; effective pixel size = 8.75 μm. Scale bar = 1 mm.

**Figure 2:**
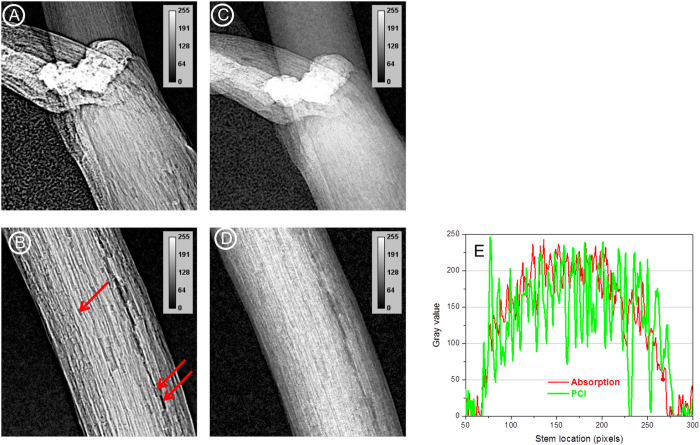
Advantages of phase contrast X-ray imaging (**A**,**B**) of plants compared to absorption X-ray imaging (**C**,**D**). The line profiles (**E**) across the middle of the plant stem (from **B**,**D**) clearly show the details of the structures evident from SR-PCI. X-ray energy used = 18 keV, exposure time = 1 s; effective pixel size = 8.75 μm. Arrows indicate different vessels in the stem.

The experimental setup used for acquiring projection (2D) images using absorption and PCI modalities at the biomedical imaging and therapy beamline-bending magnet (BMIT-BM beamline) is shown in Supplementary Figure (F1). The detector was placed ~8.5 cm (closest possible without the detector touching the plant), behind the plant for absorption imaging, and 80 cm for PCI. As the vertical size of the X-ray beam is ~7 mm, plant was rotated and then scanned vertically in steps called “slices” to record images from different parts. Figures 1 and 2 show improved image visual quality and spatially resolved structure for PCI as compared with absorption imaging. The venation system and internal structures of the cotyledon and stem of a 10 day-old canola plant were most clearly revealed by PCI. To further illustrate the advantages of SR-PCI, the stem and node sections recorded using PCI were compared with absorption images (Fig. 2). The line profiles across the middle of canola stem revealed enhanced phase contrast signals in PCI and individual vessels in the canola stem were more distinct. Distinct cavitations in the vessels (marked in Fig. 2) and structures connecting the vessels were also revealed by the PC images.

**Figure 3:**
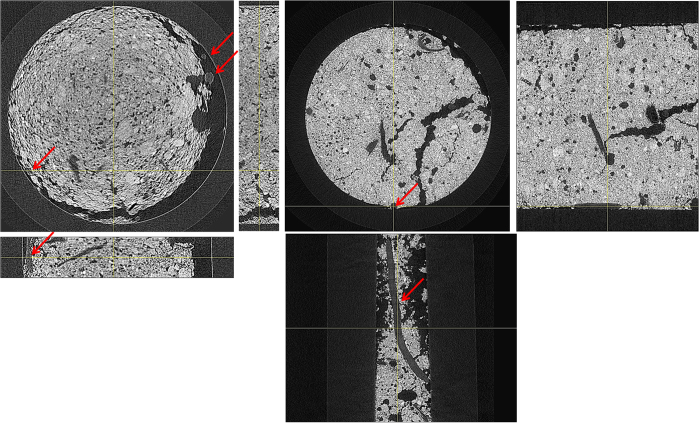
X-ray images of soil-root system recorded using SR-PCI (left, 24 keV, 5001 projection images) and laboratory (right, 50 kVp, 200 μA, 1800 projection images) X-ray imaging systems. Effective pixel size = 4.3 μm. Inside diameter of the falcon tube = 8 mm. Arrows indicate roots.

The goal is to compare SR-PCI with SR-absorption and laboratory absorption measurement for above and below ground plant samples. In the below ground samples (soil-root system), the difference between SR-PCI and SR-absorption image was not obvious and therefore compared SR-PCI of soil-root samples with laboratory absorption measurements. Figure 3 shows X-ray images of the soil-root system of a 10 day old canola plant (grown in an 8 mm diameter falcon tube) recorded using SR-PCI (24 keV, 1 frame per projection, 1 s exposure time, 60 cm from the detector) and a laboratory absorption imaging system (50 kVp, 200 μA, 2 frames per projection, 2.3 s exposure time). The effective pixel size of the images recorded using both methods was ~4.3 μm. The improved image quality of the laboratory absorption image is due to the higher X-ray energy and longer exposure time than the SR-PCI.

### Optimum X-ray energy for imaging plant seedlings

**Figure 4:**
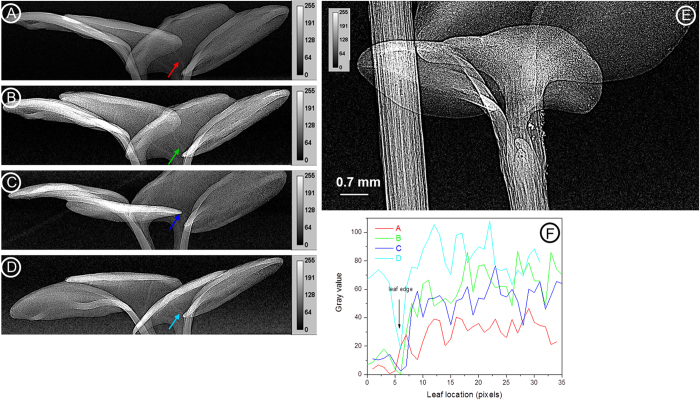
Selection of optimal X-ray energy for PCI of above ground plants. Images were recorded using an effective pixel size of 8.75 μm at 15, 18, 20 and 24 keV (**A**–**D**). The PC image of a canola plant meristem (**E**). Line profiles showing the phase signal histogram of marked regions in (**A**–**D**) (**F**). Note the movement of plant during imaging time from A to D.

The optimum X-ray energies required for above and below ground plant imaging were determined by recording a series of images at different X-ray energies (Fig. 4) and the phase signals across the region of interest have been used to optimize the X-ray energy for PCI. For above ground plant parts such as leaves, at the minimum X-ray energy (15 keV) available from the beamline the flux was low and hence the image contrast was poor. The low X-ray flux will require long exposure times. The image recorded using 18 keV X-rays had good phase contrast. As the X-ray energy was increased from 20 to 24 keV the plant became more transparent and had less phase signal (e.g. ~35% transmission in Fig. 4D). Therefore, all above ground plant parts were then scanned using an X-ray energy of 18 keV for PCI. The optimal X-ray energy for scanning soil-root systems was determined to be 24 keV for sandy clay loam soil in an 8 mm diameter falcon tube. If the plants were grown using sandy clay loam soil in an 18 mm diameter falcon tube, the required energy for optimal penetration by X-rays increased to 38 keV.

### Optimum effective pixel size

**Figure 5:**
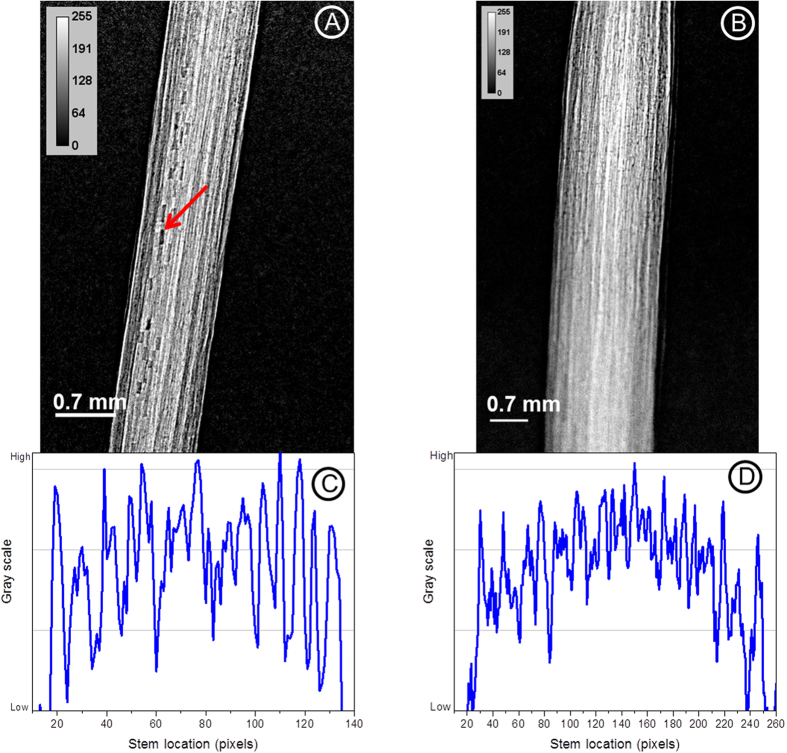
Effect of effective pixel size on plant imaging. X-ray images of a canola stem recorded at 18.0 keV using detectors with an effective pixel size of 8.75 μm (**A**) and 4.3 μm (**B**). Line profiles (**C**,**D**) across the centre of the stem from (**A**,**B**). Arrow indicates stem cavitation.

As SR X-ray flux is high, the goal is to record plant images with minimum exposure possible. The effective pixel size of X-ray images acquired using PCI depends on detector resolution and sample to detector distance^15^. Increasing the sample to detector distance increases phase signal, but decreases spatial resolution. Optimum spatial resolution required for plant imaging was determined by recording X-ray images of plants with effective pixel sizes of 8.75 and 4.3 μm (Fig. 5). Images with 8.75 μm revealed stem and leaf structures clearly and resulted in fast data acquisition. Further, all regions within the field of view were in focus compared to 4.3 μm resolution images. The 4.3 μm resolution imaging requires almost four times long exposures and can image only a narrower sample region. These attributes translate to increased radiation dose on the sample. Further, the phase contrast of the image recorded using the 4.3 μm resolution detector was low (Fig. 5D). The low contrast was evident from the line profile across the centre of a canola stem. The microstructural details of the canola stem such as cavitation, and surrounding tissues of the vessels were revealed more clearly in the 8.75 μm resolution images than in the 4.3 μm resolution images.

### Soil-root imaging

**Figure 6:**
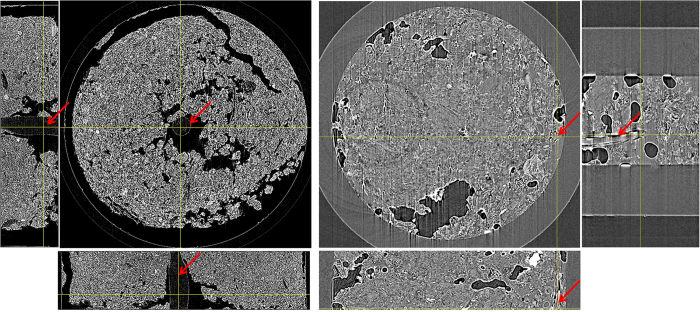
X-ray images of soil-root systems recorded using SR-PCI using sandy clay loam (left, canola seedling, 38 keV, 18 mm diameter tube) and garden mix (right, wheat seedling, 24 keV, 8 mm diameter tube) soils. Effective pixel size = 8.75 μm. Arrows indicate roots.

To determine the best soil type for soil-root imaging of plants using SR-PCI, canola and wheat seedlings grown in an 8 mm diameter flacon tube using sandy clay loam (representative of Saskatchewan soil) and planting mix were recorded at the optimized X-ray energy of 24 keV. Figure 6 shows the cross and longitudinal sections of the soil-root system extracted from CT reconstructed data sets of both soil types. The histogram profiles and visual images show that planting mix has lower contrast among soil, air space, and plant roots than the sandy clay loam soil. In the planting mix images, plant roots were often confused with organic matter in the soil. Plant roots were only realized by manual observation of CT slices from the top of the soil and by careful evaluation of root’s anatomical structures when planting mix was used. The sandy clay loam soil had distinct contrast that easily separated air space and plant roots. Further, roots did not associate closely with the soil so automated software root identification and segmentation algorithms could be used. The root system of a canola plant extracted using automated segmentation and manual interpolation between different slices is shown in Supplementary Figure F2.

### Contrast agents

**Figure 7:**
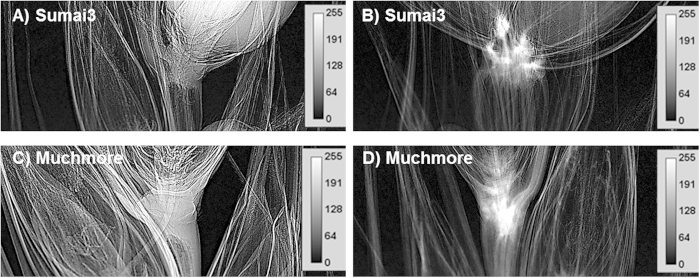
Use of contrast agents to study physiology of plants. The PCI of two wheat cultivar spikes recorded before (**A**) and after (**B**) introduction of iodine and water. The images were recorded at 18.0 keV (**A**,**C**) and 33.4 keV (**B**,**D**) using an effective pixel size of 8.75 μm.

Contrast agents have been used in SR-PCI for medical applications^28^,^29^,^30^. In agricultural science, contrast agents are used extensively in electron microscopy and X-ray imaging to highlight different parts of dehydrated flowers and leaves, and soil organic matter^20^,^8^. In this study, it has been shown for the first time that contrast agents can be used in live plants to increase image contrast to monitor dynamic processes such as water movement. In the case of wheat spikes, two cultivar spikes (Sumai3 and Muchmore) were first imaged at 18.0 keV, the energy at which wheat spikes had good phase contrast (Fig. 7A,C). The spikes were then kept in a solution of organic iodine and water for about 2.5 hours. The spikes were then imaged at 33.4 keV (100 eV above the iodine absorption edge) where the spikes had very weak phase contrast and the absorption of iodine was dominant (Fig. 7B,D). It can be seen that the movement and spread pattern of iodine was different between the two cultivars. Images of rachilla, the part of spikelet tissue that bears the florets, showed increased contrast, presumably due to more iodine bound to the tissues.

**Figure 8:**
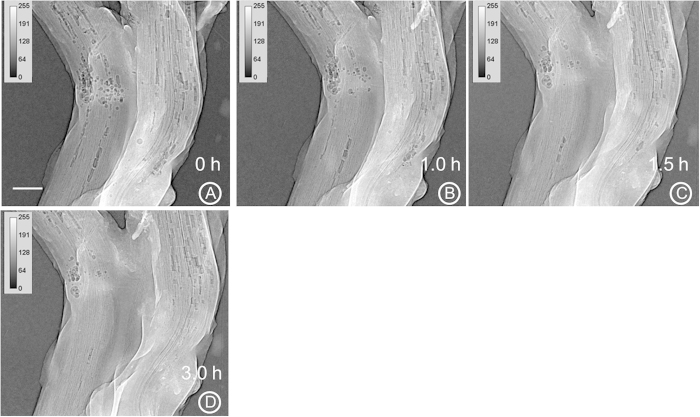
Use of PCI to determine the rate of water movement through a canola stem. X-ray image recorded before introduction of water (**A**) and series of images (**B**–**D**) recorded at different times after introduction of iodine and water. X-ray energy used was 18 keV, exposure time was 1 s and effective pixel size was 8.75 μm. Scale bar = 0.7 mm.

In another study, movement of water through canola seedling stem was observed (Fig. 8). Two 10 day-old canola seedlings were not watered for 2 days prior to being scanned to locate air cavities along the stem. It was assumed that air has entered the canola stems through roots and cavities were created in the stem vessels. The seedling roots were then immersed in a solution of water and organic iodine (1v:1v) and kept under a 60 W incandescent light while being imaged. Water movement was observed in the resulting image series (Fig. 8). The first and last images of the refilling sequence (Fig. 8A) reveal the changes in stem vessels before and after the refilling process. Some of the vessels had less phase contrast after refilling, indicating they were essentially full of water. Supplementary Figure F3 is a movie showing the disappearance of cavities as this process occurs. SR-PCI can be helpful in understanding the mechanism of cavitation and water-refilling in intact plants under water stress conditions.

## Discussion

This work explored the benefits of SR-PCI for visualization and quantification of plant internal structures and plant roots *in vivo*, under a wide range of imaging parameters to optimize data quality. In this study, we selected major crops, wheat and canola, to provide an appropriate experimental setup for SR-PCI using the BMIT-BM beamline and to compare PCI with absorption imaging (Supplementary Figure F1). The data presented here show for the first time the use of BMIT-BM at the Canadian Light Source to acquire and reconstruct 2D and 3D X-ray images of live plants (Figs 1 and 3).

It is known that imaging human carotid arteries using SR-PCI has higher SNR than a laboratory X-ray source and PCI using both systems has higher SNR than absorption imaging^31^. No such comparison is available for live plants. SR-PCI is preferred to SR and laboratory absorption imaging for studying low density plant tissues *in situ.* Further, the image quality depends on X-ray flux^21^. Laboratory X-ray imaging systems require longer data acquisition due to low X-ray flux. This study showed marked improvement in the contrast of above ground plants when images were acquired using PCI setup (Figs 1 and 2). Internal structures of canola stem were revealed in detail for the first time (e.g. visible arrangement of the cells). As shown in other works^32^,^26^, cavitation in canola stem was not continuous in the stem and filling of cavitated vessels can be visualized. Laboratory and SR-PCI systems have been used to image samples at a spatial resolution down to 100s of nm^15^,^33^. It has been also shown that SR-PCI highlighted leaf veins better than a laboratory system^13^. Further, all X-ray projection images presented here were acquired with an exposure time of 1 s per image whereas in a laboratory phase contrast imaging system it takes about 45 s to scan samples in plastic vials^19^.

The intensity of X-rays in the transmitted PC images is modulated due to edges^19^ and the intensity of the PC images cannot be directly linked to sample thickness or density. In this work, the X-ray energy to scan above and below ground seedling samples were optimized by combining the phase signals and visual image contrast. As demonstrated, the optimum X-ray energy for imaging above ground seedling parts was 18 keV with a better image quality compared to images recorded at 15, 20, and 24 keV at the same exposure time. Other reported works on imaging of bamboo and rice leaves, and grape stem have used 10–15 keV^25^,^26^. Although initial results are promising it has to be assessed further whether high X-ray energies (>40 keV) will provide images with improved contrast for visualization of internal structures of plants. A newly commissioned high brightness imaging beamline at the Canadian Light Source will be used in future studies. It’s much high flux density at high X-ray energies will likely allow a more thorough investigation of the optimal SR-PCI energy levels for above and below ground imaging.

After optimizing the energy for above and below ground imaging, the optimum phase signal distance was explored by changing the sample to detector distances. The phase signal increased when the sample to detector distance was increased from 60 to 85 cm. The highest phase signal was achieved at the maximum travel range of the sample table in the beamline (>85 cm). However, high phase signals from the edges of plant parts in the projection images (at distances higher than 60 cm) created unwanted artefacts during CT reconstruction as shown by Pratt *et al.*^34^. The exact methods for phase contrast retrieval require taking image data sets at several different distances between the sample and the detector^11^,^35^,^36^. This is very difficult with plant samples due to problems like sample motion and dehydration. Due to these reasons, such research could use only single distance phase retrieval algorithms. Unfortunately such algorithms work for samples with special properties (absorption is weak and homogenous, refractive index decrement δ and the absorption index β of complex refractive index n = 1 − δ + iβ are proportional to each other)^37^. The plants imaged in this work do not seem to be objects suitable for single distance phase retrieval^36^ (Supplementary Figure F4,4C slice of the reconstructed image without phase retrieval; 4D with phase retrieval algorithm). For the purpose of this initial study we have used only the edge enhancement properties of the phase signal selecting the distance between the sample and the detector in such a way that the interface around the features of interest has optimally enhanced edges due to the phase signal^35^.

In this study, X-ray images of soil-root system recorded using SR-PCI were compared with a laboratory X-ray imaging system. In a previously reported work, the optimal X-ray energy for root detection using SR-PCI is determined to be 25 keV^38^ with an exposure time of 2 h allowing detection of roots including branches and cross-over points. It is reported that phase contrast signals are dominant and it is possible to view some details of root anatomy when the orientation of roots are aligned with the X-ray beam. The SR-PCI has been used to study the symbiotic association of mycorrhizal fungi in plant roots by imaging washed roots and PCI is able to differentiate root hairs and fungi^39^.

In general, optimal effective pixel size required should be determined for each specific sample type in combination with specific SR technique being used. Little is known about effective pixel size required for plant imaging and the effect of pixel size on the quality of X-ray images. Although different resolution detectors are available, the selection of detector depends on the purpose of the study where the normal tendency is to use the highest resolution possible. Image pixel sizes from 10s of micron to several hundred nanometers have been used to study plant samples using PCI^31^,^18^. For example 0.7 mm and 200 nm effective pixel sizes have been used to study water transport in plants and to image roots and root hairs^40^,^33^. In this study, the advantages of low effective pixel size (8.75 μm) compared to high resolution (4.3 μm) images for plant study is shown (Fig. 5). The 8.75 μm resolution imaging permits scanning larger areas and data acquisition is faster than the 4.3 μm resolution detector. Good image quality can be obtained through optimization of a wide range of parameters such as detector resolution, X-ray energy, and sample to detector distance.

The importance of root systems for water and nutrient uptake by plants has motivated researchers to seek a better understanding of root system development *in situ* in soil. The root system may in fact hold the key to fully understanding plant growth and development, and to develop crop varieties with improved yield by optimizing nutrient use efficiency^41^. The complex root system is central to the health and survival of plants, especially when distribution of resources in soil environment is scarce and non-uniform^42^. Therefore, a good understanding of the spatial arrangement of plant root system or plant root’s architectural traits is important for studies involving water and nutrient transport, and root-microbe interactions^43^,^44^. Some research work has focused on root system complexity, but a great part of the work is done using projection images^42^,^45^. Although these studies have provided a reasonable insight into the complexity of root systems, they do not have the precision of a 3D analysis. To improve the accuracy and for good understanding of an inherently 3D structure, 3D visualization of the root systems of plants in its growth medium is crucial^44^. Soil-root interface and interactions, and root health (including root morphology, root density, and root hairs) also play an important role in nutrient uptake^33^.

Several studies have attempted to reconstruct 3D images of plant roots in natural soil with a variety of results^46^,^43^,^47^,^10^. All these studies underlined the influence of soil structure, density, and porosity on the quality of projection images. The need for further optimization of X-ray beam parameters and soil selection with lower organic matter has been emphasized. In this study, two different soil types, planting mix and sandy clay loam soil have been compared to determine the relative feasibility of extracting root architectural information and anatomical structures in each soil type. The sandy clay loam soil had good contrast between the soil and root making it easy to extract the roots with minimal effort during data analysis. Similar conclusions on the ease of sandy clay loam soil for soil-root X-ray imaging is reported by other groups^13^. Anatomical structures of the canola roots (Supplementary Figure F2) were even visible in the big roots. The results show that good quality X-ray images of soil-root system were obtained with sandy clay loam soil at optimal X-ray energy of 24 keV and 38 keV when plants were grown in 8 and 18 mm diameter tubes, respectively (Fig. 6). The optimal X-ray energy for soil root system imaging is dependent on the soil type, volume and density.

High resolution X-ray CT imaging can be used to monitor plant structures and physiological processes such as xylem network architecture and real-time water uptake^26^,^32^. Although SR-PCI creates good contrast, soft tissues of plants still have poor contrast. Iodine is the most common contrast agent used for enhancing X-ray images in biological tissues. Imaging with iodine contrast agent for medical imaging produced consistently high quality images^48^,^30^,^28^. In another study, different contrast agents used in electron microscopy were evaluated for their penetration power and effect to highlight different parts of weakly absorbing flower parts^20^. The flowers were dehydrated and infiltrated using the contrast agents. Iodine is used as a contrast agent to highlight the venation in leaves by soaking dried and cut leaves in solution^13^. As dehydration and embedding may create morphological changes and artefacts^49^,^42^, in this study it is shown for the first time that iodine can be used in freshly cut plant parts or live plants to increase the image contrast to monitor dynamic processes such as water movement.

The phase contrast images can be recorded before and just after the absorption edges of elements to highlight the elemental distributions in samples, also called K-edge subtraction technique^47^. We have used the technique to map flow and distribution of iodine in live plant samples. Different groups have combined SR X-ray CT with K-edge subtraction or by combining SR X-ray imaging with advanced X-ray fluorescence detectors such as Maia detector to study the distribution of metals and metal ions in dry plant tissues or excised fresh roots or phantoms^50^,^51^. In case of wheat spikes, the movement and spread pattern of iodine was different in the two examined cultivars (Sumai3 and Muchmore) at 33.4 keV (Fig. 7).

The results from the water rehydration study clearly show the water refilling of canola stem cavitation when exposed to iodine and water (Fig. 8). The PCI clearly showed both plant anatomy and transport of iodine-water within the xylem vessels. So far, very few studies have determined water refilling using SR-PCI in plants and most of them did not use contrast agents^52^,^26^,^53^. While the precise mechanism for water-refilling is still not fully understood, it is clear the SR-PCI is deemed to be a powerful, high resolution, real time imaging tool to investigate the water-refilling process in xylem vessels in plants^52^. Iodine when used in high concentration (1v:1v) with water, was damaging tissues like leaves in canola after exposure for an extended period of time. Future work will focus on optimizing the concentration of iodine to minimize the effect on plant tissues. Low concentrations of iodine may not be harmful to plants as iodine is used for bio-fortification in vegetable plants^54^.

The computed tomographic implementation of emerging X-ray transmission imaging modalities, such as SR absorption and PCI present important applications to biology and medicine^55^,^56^,^57^,^58^,^59^,^60^,^23^. Recently, numerous studies have highlighted the use of SR-PCI for 3D visualization of internal structures as a minimally invasive and fast imaging technique^20^. As large-scale and compact synchrotron facilities are currently under rapid development worldwide^61^, the implementation of PCI with CT could find broad applications in plant biology. The use of these advanced synchrotron X-ray facilities can be used to study and understand the complexity of the internal structures and biological phenomena in *planta* in great detail.

One of the challenges of imaging above ground plant parts *in vivo* is the plant movement due to environmental factors. Therefore, lots of published works on above ground plant are done on excised or dry plant parts such as cut bamboo or dry leaves^25^,^26^. The movement of plants was also realized during imaging in this study (Fig. 4A–D) if the plant was left in open air. The high flux in the SR makes it possible to image fast, however plant movements sometimes created problems during 3D CT reconstructions. To minimize the problem of plant movement during imaging (due to dehydration or air currents), a falcon tube was used to cover and as a support to plant structures such as leaves or flowers. Using this procedure we have successfully reconstructed 3D images of some above ground plant parts like leaves *in vivo* (Supplementary Figure F2) and canola flowers (Supplementary Figure F4). Further technical advances are necessary to minimize plant movement for optimal image acquisition.

### Radiation damage

Although the image quality is greatly enhanced using SR-PCI compared to conventional PCI, SR has high flux that translates to the amount of radiation (or heat load) the plant is exposed to, and hence plant tissues may be damaged. The radiation damage from SR imaging is still lower than conventional systems due to fast imaging and use of monochromatic beams at the SR limits the exposure of plants to unwanted low energy X-ray beams (due to the wide bandwidth in conventional systems). For a broadband X-ray source, the low energy X-rays typically do not contribute significantly to the image, while providing the majority of the absorbed dose to sample. The unwanted X-ray beams that are not useful for acquiring images may contribute more to the sample damage^15^. Some condensation on the inside walls of falcon tubes in which the plants were held was observed during this study if the imaging session was more than an hour. The effect of radiation damage on plant tissues has been demonstrated by combining X-ray imaging with chlorophyll fluorescence imaging of cut rose peduncles^62^. Browning effect and structural damage is observed and it has been shown that regions exposed to X-rays have reduced chlorophyll activity when the images are recorded in high flux beamline using superconducting 7T wavelength shifter as the source. In this study, no immediate browning effect of the tissues was observed similar to other reported work as the source used in this study is a bend magnet^63^. Further, high energy X-rays are known to exhibit less radiation damage in biological samples compared to low energy X-rays^62^.

## Conclusions

We have developed, for the first time at the Canadian Light Source, novel methodologies for using SR-PCI for better understanding of complex internal plant structures and dynamic processes. The SR-PCI technique is a minimally invasive technique that could be used successfully to reconstruct internal structure of plants in 3D using micron- to nano-scale resolutions. The technique is very useful to understand dynamic processes by monitoring the plant states at different conditions of abiotic, biotic, or nutritional stresses. Furthermore, it is expected that the relatively low radiation dose absorbed by the samples will, in future, allow longitudinal studies in which the same living plant can be imaged in a series of time separated exposures. The image qualities presented in this work are better than some of the earlier reported works. The beam parameters used in this work can be improved by dedicated beamlines optimized for plant research. The work illustrates proper selection of soil types, effective pixel size, X-ray energy, and sample to detector distances are required for high quality plant images.

## Methods

### Plants

The canola (*Brassica napus*, cultivar Fortune RR), and wheat plants (*Triticum aestivum* L., cultivars Sumai3 and Muchmore) used in the experiments were grown under controlled conditions using a plant growth cabinet (18–23 °C) with 14-h photoperiods (512 μmol.m^−2^s^−1^). The seedlings were transported to the beamline for imaging a few hours prior to beamtime and were kept in a well-lit room until the data was collected. Wheat spikes of Sumai3 and Muchmore were excised from plants in the growth chambers 4 hours prior to beamtime and were kept at room temperature inside air-tight freezer bags. The seedlings were grown using soil-less planting mix (pH 5.8–6.2, Sunshine #3, SunGro Horticulture, Vancouver, BC) for above ground imaging experiments. For soil-root X-ray imaging, soil-less planting mix and sandy clay loam^64^ soils were used to compare the merits of different soils for recoding X-ray images.

### Synchrotron-based phase contrast X-ray imaging

The phase contrast X-ray imaging data of all plants were collected at (BMIT-BM, 05B1-1) at the Canadian Light Source^65^,^66^. The X-ray source of the beamline is a bend magnet in a 2.9 GeV synchrotron^62^. The accessible energy range of the beamline is 18–40 keV and the beamline produces a monochromatic and coherent X-ray beam of size 240 mm (horizontal) × 7 mm (vertical) into the experimental station, which is about 23 m from the bend magnet source. The optimum X-ray energy for SR-PCI of above ground and below ground plants was determined by comparing the phase signals from the transmission X-ray images of the samples. Two, 0.5 and 1.0 mm thick aluminum filters were used before the monochromator when the energy was below or above 20.0 keV, respectively to reduce the heat load on the monochromator. The transmitted X-ray images were converted into visible images by a combination of scintillator and a visible camera (called a detector). An 8.75 μm effective pixel size (C9300-124, Hamamatsu, 35 mm diameter, AA-60) was used for most studies and a 4.3 μm effective pixel size (C9300-124, Hamamatsu, 14 mm diameter) was also used to determine the effect of image resolution for plant imaging. All projection images collected were corrected for the dark signal from the detector (without X-ray beam) and flat signal (with X-ray beam and no sample) due to imperfections in the beam and inhomogeneity in the scintillator screens. For the CT data sets, 10 images of flat and dark signals were recorded before and after recording the CT images and the average of the flat and dark images were used for normalization^11^.

### Laboratory based absorption X-ray imaging

To determine the advantages of synchrotron based SR-PCI for soil-root imaging, a 10 day-old canola plant first imaged by SR-PCI was then scanned using a laboratory X-ray machine (SkyScan1172, Bruker microCT), as the difference of absorption and phase contrast images collected using the synchrotron SR-PCI did not show clear differences compared to above ground plant parts. The image contrast based on the histogram distribution, phase signal, visual image quality, and data acquisition time between SR-PCI and laboratory X-ray imaging were compared.

### Contrast agents for plant imaging

Iodine is used as a contrast agent in medical imaging to improve the contrast in images. The use of iodine to improve the contrast in plant vessels such as xylem to study plant physiology and process dynamics in live plants was determined in this study. The SR-PCI of plants were first recorded at an X-ray energy of 18 keV and then the plants with roots (with soil washed out) or wheat spikes were inserted into a solution of water and organic iodine 1:1 (v/v) (Optiray^TM^ 240, Ioversol Injection 51%). Plant parts were then imaged using X-rays at or just above the absorption energy of iodine at 33.4 keV.

### Water movement in plant stem

To create cavitation in plants, 10 day-old canola seedlings were not watered for 2 days prior to beamtime. The seedlings were first scanned at an X-ray energy of 18 keV to locate air cavities in the stem. The plant roots were then immersed in a 1:1 (v/v) solution of water and organic iodine and plants were kept under a 60 W incandescent light to increase the transpiration rate. The canola stem was imaged at the same X-ray energy every 10 min for about an hour and once every 15 minutes after the first hour.

### Data analysis

The projection X-ray images were first normalized using flat and dark signal images (ImageJ 1.44p, National Institutes of Health, USA). The 3D reconstruction of images was carried out using NRecon software (version 1.6.9.4, Skyscan) and 3D data analysis (segmentation etc.) was accomplished using Avizo standard software (version 7.1.1, Visualization Sciences Group).

## Additional Information

**How to cite this article**: Karunakaran, C. *et al.* Factors influencing real time internal structural visualization and dynamic process monitoring in plants using synchrotron-based phase contrast X-ray imaging. *Sci. Rep.* **5**, 12119; doi: 10.1038/srep12119 (2015).

## Supplementary Material

Supplementary Information

Supplementary Movie 1
