# Supplementary material for: Factors influencing real time internal structural visualization and dynamic process monitoring in plants using synchrotron-based phase contrast X-ray imaging
Source: Sci Rep. 2015 Jul 17;5:12119. doi: 10.1038/srep12119 (PMC4648396; doi:10.1038/srep12119)
Supplement: Supplementary Information [file srep12119-s1.pdf]

Factors influencing real time internal structural visualization and dynamic process monitoring in plants using synchrotron-based phase contrast X-ray imaging

Chithra Karunakaran[1*], Rachid Lahlali[1], Ning Zhu[1], Adam M. Webb[1], Marina Schmidt[2], Kyle Fransishyn[2], George Belev[1], Tomasz Wysokinski[1], Jeremy Olson[1], David M.L. Cooper[3], Emil Hallin[1]

[1]Canadian Light Source Inc., 44 Innovation Boulevard, Saskatoon, SK, Canada S7N 2V3
[2]University of Saskatchewan, 110 Science Place, Saskatoon, SK, Canada S7N 5C9
[3]Department of Anatomy and Cell Biology, University of Saskatchewan, 107 Wiggins Road, Saskatoon, SK, Canada S7N 5E5

*****Corresponding author: Email: Chithra.Karunakaran@lightsource.ca, Phone: +1-306-657-3749, Fax: +1-306-657-3535

Supplementary Figure F1

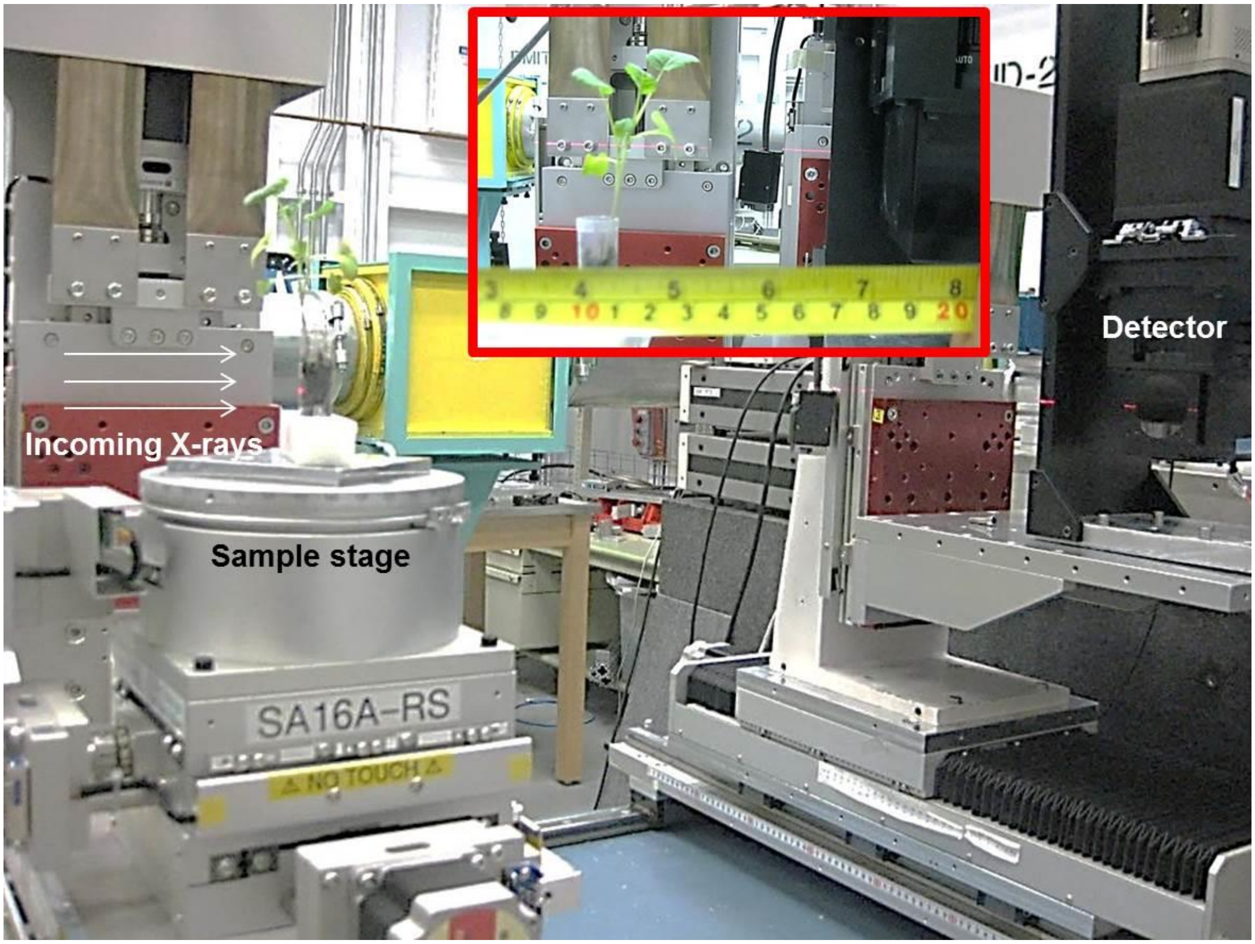

Supplementary Figure F1. Experimental setup for phase contrast X-ray imaging of plants at the BMIT-BM beamline. The plant (canola, about 2 weeks old) was kept at ~85 cm away from the detector. Inset shows the setup similar to absorption imaging where the plant was moved to ~8.5 cm in front of the detector.

Supplementary Figure F2

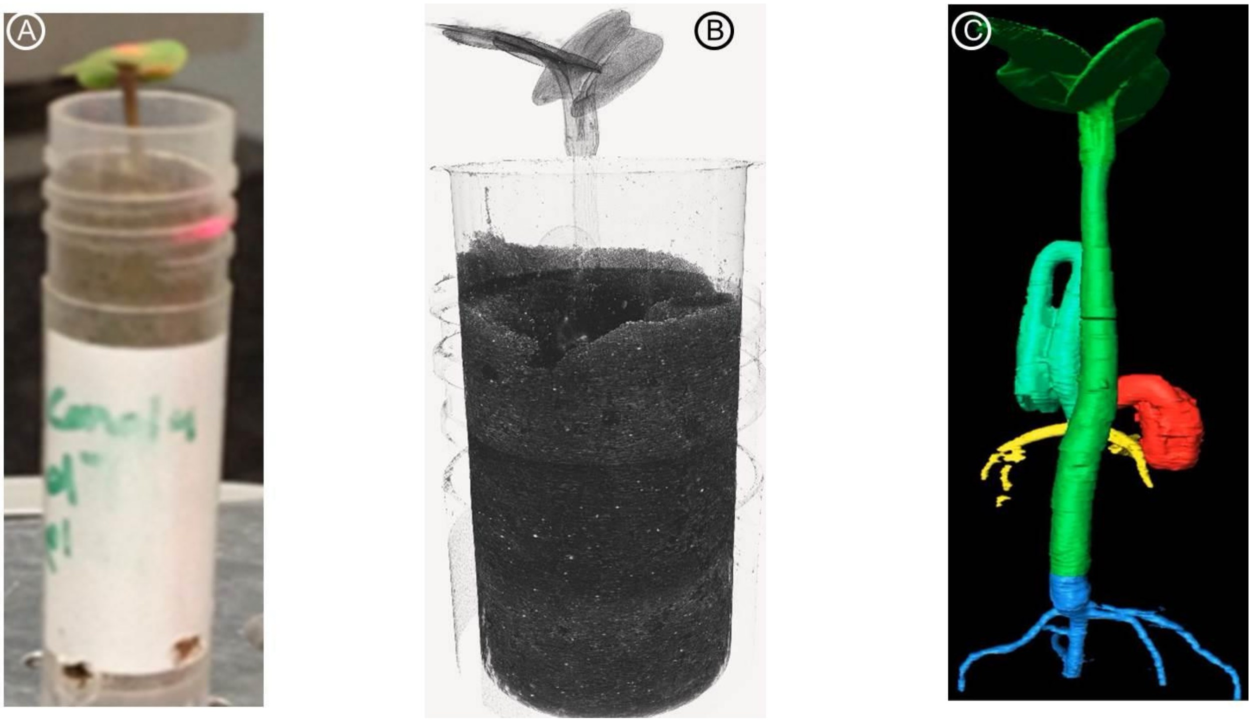

Supplementary Figure F2. SR-PCI of canola plant-root system. A) Soil-plant prepared for X-ray imaging; B) Reconstructed X-ray image; and C) Movie of the segmented canola plant.

Supplementary Figure F3

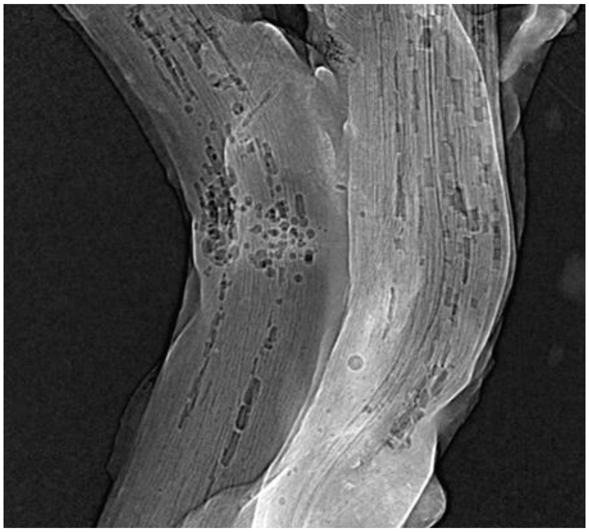

Supplementary Figure F3. Movie of water movement through canola plant stem.

Supplementary Figure F4

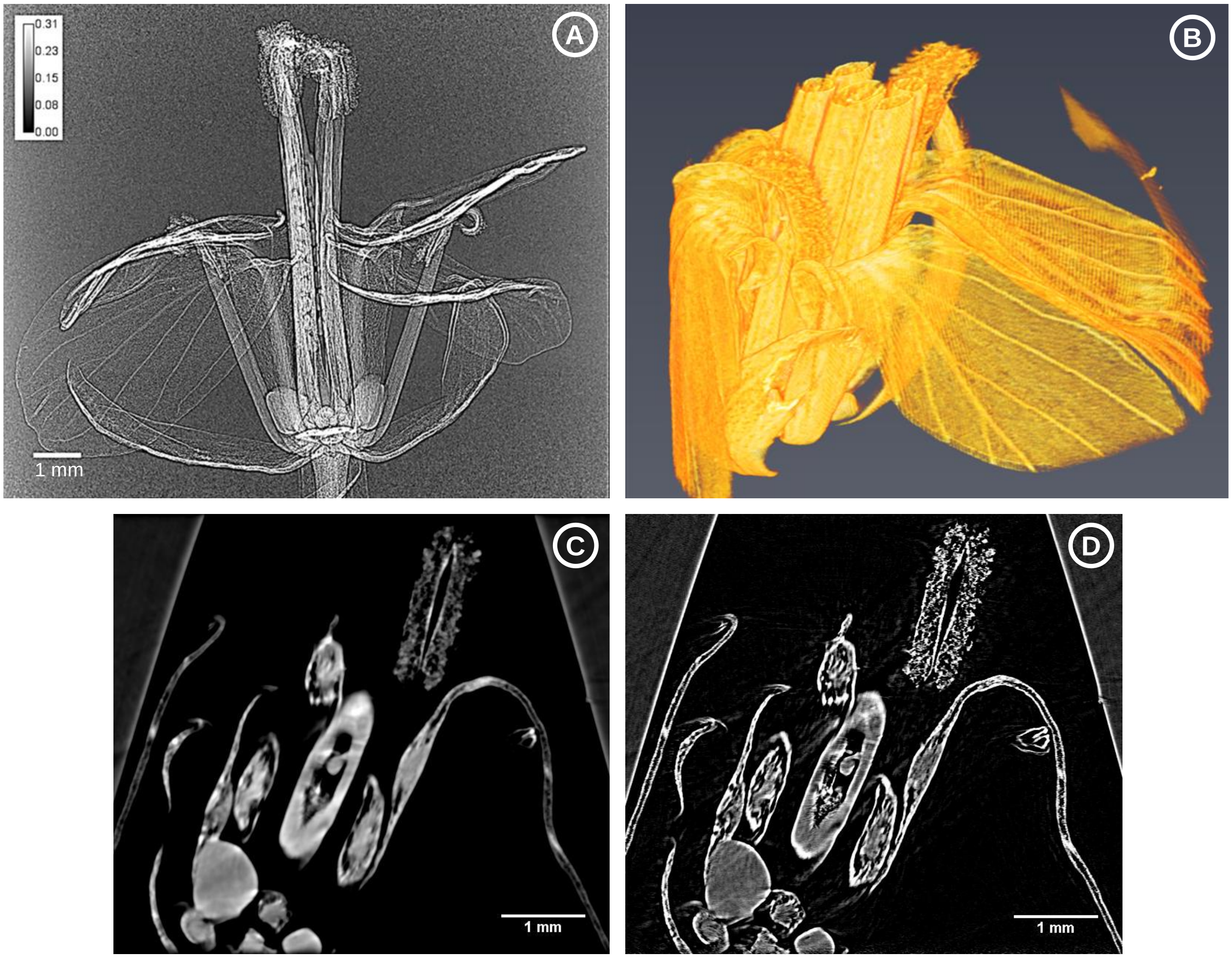

Supplementary Figure F4. The SR-PCI of canola flowers. A) 2D image; B) 3D reconstructed image; C) 2D image slice from a reconstrcued data set obtained after using a phase retrival algorithm; D) 2D image slice from a reconstrcued data set obtained without using any phase retrival algorithm.
