# Supplementary figures and images for: Factors influencing real time internal structural visualization and dynamic process monitoring in plants using synchrotron-based phase contrast X-ray imaging
Source: Sci Rep. 2015 Jul 17;5:12119. doi: 10.1038/srep12119 (PMC4648396; doi:10.1038/srep12119)

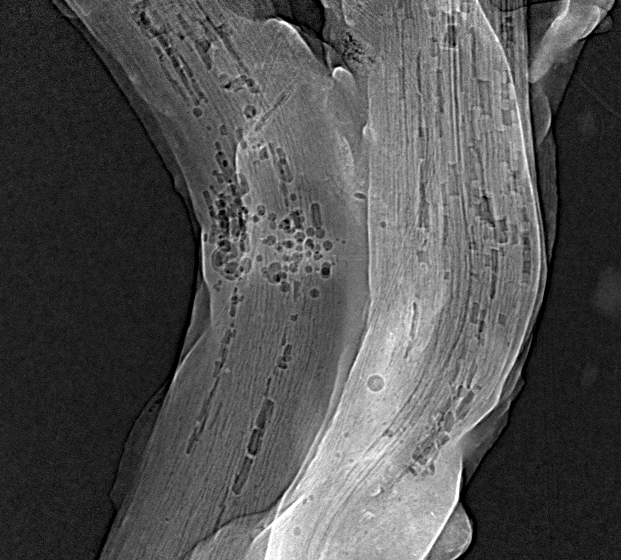

Supplement: Supplementary Movie 1 [file srep12119-s2.gif]
